# Determinants for further wishes for cosmetic and reconstructive interventions in 1652 patients with surgical treated carcinomas of the oral cavity

**DOI:** 10.1186/s40902-017-0125-1

**Published:** 2017-09-05

**Authors:** Henrik Holtmann, Simon Spalthoff, Nils-Claudius Gellrich, Jörg Handschel, Julian Lommen, Norbert R. Kübler, Gertrud Krüskemper, Majeed Rana, Karoline Sander

**Affiliations:** 1 Department for Oral and Maxillofacial Surgery, Heinrich Heine University of Düsseldorf, Moorenstr 5, 40225 Düsseldorf, Germany; 2 Department of Cranio-Maxillofacial Surgery, Hannover Medical School, Carl-Neuberg-Street 1, 30625 Hannover, Germany; 3 Department of Medical Psychology, Ruhr University of Bochum, Universitätsstr 150, Building MA 0/145, 44780 Bochum, Germany

**Keywords:** Cosmetic surgery, Scarring, Quality of life, Depression, Coping, Oral cancer

## Abstract

**Background:**

The impairment of the appearance is a major problem for patients with carcinomas of the oral cavity. These patients want to recover their preoperative facial appearance. Some do not realize that this is not always possible and hence develop a desire for further cosmetic and reconstructive surgery (CRS) which often causes psychological problems.

**Method:**

The desire of patients for CRS (*N* = 410; 26%) has been acquired in this DÖSAK rehab study including multiple reasons such as medical, functional, aesthetic and psychosocial aspects. They relate to the parameters of diagnosis, treatment and postoperative rehabilitation. Patients without the wish for CRS (*N* = 1155; 74%) served as control group. For the surgeons, knowledge of the patient’s views is relevant in the wish for CRS. Nevertheless, it has hardly been investigated for patients postoperatively to complete resection of oral cancer. In this retrospective cross-sectional study, questionnaires with 147 variables were completed during control appointments. Thirty-eight departments of Oral and Maxillofacial Surgery took part, and 1652 German patients at least 6 months after complete cancer resection answered the questions. Additionally, a physician’s questionnaire (*N* = 1489) was available. Statistical analysis was performed with SPSS vers. 22.

**Results:**

The patient’s assessment of their appearance and scarring are the most important criteria resulting in wishes for CRS. Furthermore, functional limitations such as eating/swallowing, pain of the facial muscles, numb regions in the operating field, dealing with the social environment, return to work, tumour size and location, removal and reconstruction are closely related.

**Conclusion:**

The wish for CRS depends on diverse functional psychosocial and psychological parameters. Hence, it has to be issued during conversation to improve rehabilitation. A decision on the medical treatment can be of greater satisfaction if the surgeon knows the patients’ needs and is able to compare them with the medical capabilities. The informed consent between doctor and patient in regard to these findings is necessary.

## Background

Early on surgeons recognized the importance of psychological variables for coping with distortions in the head and neck area [1–3]. However, the conditions for the implementation of the findings into rehabilitation were not given at that time [4]. Even more not only somatic but also psychological factors for patient satisfaction are important [5–8].

In a multipart work, Kollbrunner determined psychological variables in 2001 [9, 10]. Apart from survival, quality of life (QOL) targeting on dealing with functional and aesthetic impairments became more important in the last 15 years. Nowadays, finally, fundamentals for interdisciplinary care of patients after head and neck surgery are acquired [11–13].

In recent studies, quality of life is defined differently taking disease-specific variables into account (health-related quality of life (HRQL)).

The aim of this study was to define the experience of impairment by asking the patients and emphasizing on aspects of disfigurement and the desire for further cosmetic and reconstructive surgery [14]. Personality traits, psychosocial factors and coping strategies as well as the effects of support, coping and resilience [15] have been identified.

A particularly important coping strategy is the patient’s endeavour to regain an appearance as close as possible to the preoperative state. This is an illusion, well known to the maxillofacial surgeon but not to the patient. However, there is hope to increase the satisfaction in patients making compromises. Accordingly, the patient needs to be given informative support. Until now, this has hardly been taken up in literature. Few is known describing the importance of scarring and disfigurement of the patient and emphasizing on the importance of quality of life.

The consequence to require further cosmetic surgery has not been researched, even though it is meaningful to maxillofacial surgeons [16]. However, even more significant is the discrepancy between desires of patients to improve their situation and the offer made by the medical and psychological side.

Millsopp [16] discovered significant differences in the causes for further cosmetic and reconstructive surgical wishes in patients. While 114 (41%) of 278 patients hoped to improve their situation by CRS, only seven of the medical reports portrayed a dissatisfaction with postoperative appearance and only in two cases further CRS was described as necessary.

**Fig. 1:**
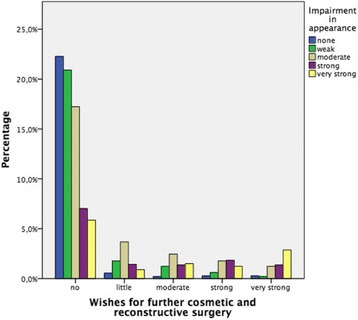
Impairment of appearance grand wish for further CRS

In this study, the relationships between the desire for cosmetic surgeries and its determinants are analysed (Fig. 1).

This should help to rehabilitate the patient after radical surgical removal of oral cancer, to improve the subjective satisfaction and hence the quality of life. Whether another operation makes sense can be determined based on the knowledge of medical data and the patients’ desires. A further aim of this study is to introduce the most significant data needed in the interview between doctor and patient, in order to plan further operations in maxillofacial surgery according to the patient’s needs.

In recent studies, an extended term of life quality (LQ) is being used, describing the connection between surgical techniques and health-related quality of life (HRQL) [17, 18]. Hence, insights on interactions between impairments reported by patients were collected. In addition, an attempt was made to explore the psychosocial conditions or consequences of those impairments [19–26]. At times, no significant differences were found for general LQ, but often for HRQL [22–24, 27, 28].

## Methods

In this retrospective study, 3894 questionnaires were handed out to German-speaking patients by oncological wards in 43 participating hospitals in Germany, Austria and Switzerland. Of those 1761 returned anonymously in time [29], 1652 were evaluable. Nine chapters with 147 complex questions gathered demographic data, health behaviour, diagnosis and treatment prior and during the in-patient-stay. Furthermore, the development of the impairments caused by disease and therapy for at least 6 months after surgery was analysed. The questionnaire was developed by the Oral- and Maxillofacial Surgery and the Department for Medical Psychology of the Ruhr-University Bochum and tested for systematic and unsystematic mistakes (Thesis Grochowski & Hendler, 1993 unpublished). Thirty-eight of the 43 participating hospitals returned the questionnaires. Tumour size was specified in 1489 questionnaires. Answers to life quality were not answered in 149 cases. Furthermore, not all patients answered all questions which resulted in minor differences in sample size.

To measure the experienced impairment, a 5-step Likert scale was used (no impairment = 0, slight impairment = 1, moderate impairment = 2, severe impairment = 3, very severe impairment = 4). The result figures relate to the time before treatment (t1), immediately after (t2) and at least 6 months after surgery (t3). The quality of life was evaluated via a 100-ary scale (from 0 = not satisfied to 100 = completely satisfied). The psychological variables were measured by the following scales:

- Depression: von Zerssen Depression Scale [30]
- Anxiety with STAI: State-Trait Anxiety Inventory [31]
- Coping with the FKV: Freiburg Questionnaire of Coping with Disease [32]
- The locus of control with the IPC-scales on locus of control [33] KKG in the German version and abbreviated form

Statistical analysis uses differences calculated by SPSS 22. Occasionally, standard residuals (SR) are mentioned in parenthesis. If significant, differences between groups are emphasized by SRs of ≥2 in crosstabs relating to the subgroups. The calculation of significant differences was performed according to Kruskal-Wallis with a univariate ANOVA second to using the Komologov-Smirnoff test. Significances in correlations and cross tabulations were calculated according to Kendall’s tau b. In addition, linear stepwise regression was used. The results are indicated in boxes.

## Results

In 1565 of the 1652 evaluable questionnaires, the request on additional cosmetic and reconstructive surgeries was answered. Eighty-seven patients took no position (missing 5%). In 74% of the sample, there was no desire for another cosmetic surgery. Twelve percent of patients expressed a strong and very strong desire for further cosmetic surgery, and 14% of this desire was moderately or very pronounced (Table 1). Hence, 26% of patients would need a conversation on this topic.

**Table 1:** Wish for further cosmetic surgery

		Frequency (n)	Validity (%)
Valid	none	1155	74
	little	127	8
	moderate	102	6
	severe	90	6
	very severe	91	6
	Total	1565	100.0
Missing	System	87	
Total		1652	

One thousand five hundred eighty patients answered the question on the number of underwent cosmetic surgeries (missing 74, 4%). Ninety percent of the sample did not have any cosmetic surgery (*N* = 1417). One hundred twenty-five people (8%) were operated once cosmetically, 16 (1%) twice and 22 (2%) more than two times. Frequencies for localization are shown in Table 2.

**Table 2:** Tumour localization (number of patients counted (*n*) = 1484

Localisation	Percentage
Floors of the mouth (144)	42
Tongue (141)	25
Alveolar gingiva (143)	17
Other nonspecific sites (145)	16

### Sociodemograhic data

There was no significant statistical difference between men (*n* = 1239) and women (*n* = 413) (*p* = 0.12) regarding the wish for CRS.

In contrast, the younger patients aged 14–45 years (*N* = 131) varied significantly (−0.196** reg. Kendal’s tau-b) as compared with older patients (*n* = 1459). Patients aged above 45 years seldom wished for further CRS.

The family status was found to be important as single or divorced/separated patients more often had a strong wish for further cosmetic and reconstructive operations (*p* < 0.003 SR 2.4; 1.8 cross tabulation).

No further significant results were found in between the different educational levels (*p* < 0.208 cross tab). Even the highest vocational graduation did not significantly influence the wish for further CRS (*p* < 0.411 cross tab) whereas significantly strong wish occurred in context of the current professional career (*p* < 0.001 cross tab). Hence, 202 patients who passed the age limit of pension negotiated the wish for CRS more often. In contrast, 66 patients, who were prematurely retired due to tumour operation, were strongly interested in further CRS (very strong SR 3.8 and strong wish SR 1.9). Religious confession was not relevant, but there was a high and significant wish for CRS in patients with more than 500 € loss of income.

Furthermore, the patients’ satisfaction with their appearance was influenced by the postoperative scarring (Table 3): Only 10% of the examined patients stated no significant facial scarring. This fact is due to the tumour localisation. Forty percent suffered from excessive or very excessive cicatrisation; 48% mentioned minor or moderate cicatrisation. Nevertheless, by relating those numbers with the wish for further CRS, a total of 74% did not want to undergo further cosmetic surgery.

**Table 3:** Felt severity of scar formation by patients

Severity of scar formation	Number	Valid percentage
None	162	10
Sparse	258	16
Moderate	516	32
Strong	420	26
Very strong	260	16
Valid	1616	100
Missing	36	
Entire	1652	

**Fig. 2:**
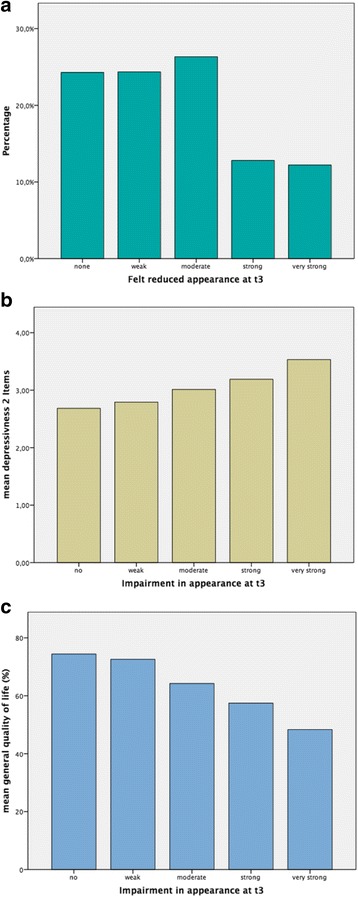
Appearance at t3: **a** number and percentage of affected patients, **b** relation to depressiveness, and **c** relation to life quality

Patients evaluated their impairment in appearance at three different points in time as mentioned before (Table 4). Altogether, 19 impairments were found in fields of oral cancer surgery and are listed in Table 7. Only few patients pre-operative to surgery (t1) mentioned impairments in appearance. Immediately after surgery (t2) and possibly after additional radiotherapy, negative experiences were seen concerning strength and number of cases. Sixth months after surgery, those experiences wear off/ease without ever reaching the pre-operative state again. Different manifestations of this impairment were found in 1153 of 1652 patients (t3). Stronger manifestations lead to significantly lower life quality (48 of 100% in Fig. 2c). Simultaneously, depressiveness increases significantly from 2.7 to 3.6 points according to v. Zerssen Depression Scale (Fig. 2b).

**Table 4:** Typical chronological process of impairments using the example of appearance

Impairment	*n* t1^a^	Percentage	*n* t2^a^	Percentage	*n* t3^a^	Percentage
None	1161	78	225	15	370	24
Sparse	131	8.8	228	15	371	24
Moderate	99	6.7	340	22	401	26
Strong	49	3.3	333	22	195	13
Very strong	42	2.8	410	27	186	12
Entire	1482	100	1536	100	1523	100

^a^t1 pre-operative to surgery; t2 immediately after surgery; t3 6 months postoperative: 1153 patients reported impairments of different strength; *n* = number of patients)

### Medical data

The wish for further CRS was more frequent in patients with bilateral affected localisations (*p* < 0.001). A notable correlation is also to be found in tumour stage and wish for CRS (*p* < 0.001): Patients with pT1 staged tumours negated the wish more often than patients with pT4 tumours, who strongly (SR 2.1; *N* = 20) or excessively (SR 3.9; *N* = 27) longed for CRS. The correlation of lymph node metastases and the wish for CRS was also highly significant: patients with pN2 stage longed more often for further CRS (*p* < 0.001) as well as patients with pN3 stage. On the contrary, most patients without lymph node metastases did not want further surgery (*N* = 659 of 862). Patients who underwent surgery and radiotherapy (*N* = 608) were determining for the significant difference in wishes for further CRS (*p* < 0.001).

Table 5 shows the number and type of reconstructions which were carried out after surgical treatment of oral cancer. Nearly 50% of the patients were reconstructed using local tissue. Table 6 shows the relationship between neck dissection/reconstructions and the further wishes for CRS, felt appearance und felt cicatrisation.

**Table 5:** Frequency and type of reconstruction after surgical treatment of oral cancer

Reconstruction	Count	Percentage
No reconstruction	102	6.2
Only microvascular flaps	202	12.2
Only pediculated flaps	261	15.8
Pediculated and microvascular flaps	20	1.2
Only local tissue	798	48.3
Local tissue and microvascular flaps	23	1.4
Local tissue and pediculated flaps	120	7.3
Local tissue and pediculated and microvascular flaps combined	6	0.4
Missing	120	7.3
Entire	1652	100

**Table 6:** Relation between neck dissection/reconstructions and the wish for CRS (A), felt appearance (B) and felt cicatrisation (C)

Neck dissection (ND) and reconstruction	A	B	C
Suprahyoidal ND	n.s.	0.037	< 0.001
Radical ND	< 0.001	< 0.001	< 0.001
Functional ND	0.008	0.008	0.008
Local tissue	0.017	< 0.001	< 0.001
Pediculated flap	n.s.	0.002	0.002
Microvascular flap	0.004	< 0.001	< 0.001
Bony lower jaw reconstruction	< 0.001	< 0.001	< 0.001

*n.s.* not stated

### Impairments

Figure 1 shows the percentage of patients who longed for further CRS. Even though some patients most intensively longed for CRS (yellow), however, nearly half of all patients do not want any. Last-named belong to the group of older and socially grounded persons as mentioned above. Expectably patients who intensively suffer from impairments had an intense wish for further surgery.

In the group of t3 (6 months after surgery), 7 of the 19 impairment types were statistically significant in relation to the wish for further CRS (*p* < 0.01). The ranking order reads as follows:

1. Appearance (cor.280)
2. Mandibular mobility (cor.151)
3. Tongue mobility (cor.129)
4. Force condition (cor.127)
5. Opening of the mouth (cor. 106)
6. Swelling (cor. 99)
7. Speech to foreigner (cor. 92)

and refers to 410 patients who at least mentioned a mild wish for CRS. All remaining other impairments were not statistically related to the wish for CRS. The linear stepwise regression analysis shows that appearance (*F* = 35,609) and secondly mandibular mobility (*F* = 18,086) at t3 are the most significant variables in developing a strong wish for CRS. As a matter of fact, those two constants are so important that other impairments do not give further explanations (impact variable: (constant), tongue mobility, cervical mobility, speech to foreigners, swelling, force, mouth opening; depending variable: wish for further CRS).

More medical data is gained via the patient-questionnaire concerning diet, cicatrisation, functionality of the facial muscles, numbness and pain in the operating field. Significant differences were calculated using Kruskal-Wallis after analysis with the Komologov-Smirnoff test and univariate ANOVA.

Those 410 patients with wish for further CRS significantly differ from the 1155 patients (missing = 87) who were not interested in further CRS concerning the variables listed in Table 7. The *F* value is vast regarding the variable “liquid diet” and highly significant. This fact illustrates that diet seems to be of major importance for the development of wishes for CRS. Hence, it cannot be accidental and emerges because of discontent with liquid diet.

**Table 7:** Parameters affecting the wish for further CRS (Kruskal-Wallis)

Impairment of…	Chi-square Pearson*	Wish rank for CRS/*n* of 410
Appearance (cosmesis)	234,569	1/*N* = 392
Mobility of the mandible	95,389	2/*N* = 389
Mobility of the tongue	82,461	3/*N* = 390
Mobility of the neck	80,990	*N* = 386
Mouth opening	79,724	5/*N* = 391
Speech to foreigner	74,179	7/*N* = 386
Eating/swallowing	73,335	*N* = 395
Speech to relatives	68,379	*N* = 396
General condition	54,562	4/*N* = 393
Mobility of the shoulder	51,046	*N* = 389
Swelling	45,490	6/*N* = 383
Halitosis	41,925	*N* = 383
Taste	37,025	*N* = 388
Pain	29,690	*N* = 388
Gastric disorders	28,033	*N* = 386
Appetite	26,624	*N* = 388
Dryness of the mouth	20,811	*N* = 388
Smelling	14,149	*N* = 389
Breathing	13,361	*N* = 381

*Statistical significant ranking order *p* < 0.01

It was asked for normal oral diet as countercheck which reflects the satisfaction of patients not wishing for CRS. Scarring of the face and neck is the main trigger (*F* = 33,779; *p* < 0.001) of wishes for further CRS. Therefrom, impairments of the facial muscles lead to dropped mouth corners, and hence, dripping of saliva or drinks is the most important. If facial muscles are unaffected (*F* = 48,026 in control group), the wish for CRS is much more seldom. Hypaesthesia of the lower lip is also meaningful emphasized by the control group with significantly lower numbers of wish for CRS (*p* < 0.001). Same is valid for patients without facial or cervical pain (*F* = 12,378).

Loss of teeth is also of major importance in the development of wishes for further CRS. There is a significant difference (*p* < 0.001) between patients who lost their teeth and those who did not. The wish for further CRS grows with increasing loss of teeth (especially in the lower jaw).

### Test psychological variables

#### Questionnaire of illness processing (FKV1)

The shortened questionnaire by [32] showed that patients wishing for CRS (*N* = 410) and patients not doing so (*N* = 1155) significantly varied in terms of FKV1. Standard residuals (SR) from Chi-squared test (Pearson) show that these significant differences belong to the fact that patients with strong wish for CRS as well show high values for FKV1.

#### FKV 2 “depressive illness coping”

Standard residuals illustrate significant differences between the groups of patients longing for CRS (*F* = 410): Those who strongly wish for CRS as well show increasing depressive coping strategies.

#### FKV 3 “hedonism”

Here too, minor wish for CRS is related to minor hedonism. Hence, if the value for hedonism increases, so does the wish for CRS. Patient numbers in higher factor values are quiet small. In severe illnesses, thoughts of life enjoyment are seldom.

#### FKV 5 “mistrust and pessimism”

A strong wish for CRS is related to high levels of mistrust and pessimism.

#### Locus of control in illness and health (KGG)

Of the three factors, only factor 1 shows significant differences between the groups which varyingly strong wish for CRS (*N* = 410): Patients with increasing wish for CRS more often react internal.

#### Loci of control (IPC) by Krampen

Patients with wishes for CRS more often think that others should be helping.

#### Depressiveness scale by Zersson

The difference between patients without (*n* = 1155) and those with (*n* = 410) wish for CRS is statistically significant (*p* < 0.001, chi-squared). Standard residuals describe the tendency that an increasing wish for CRS comes along with increasing depressiveness.

#### State trait anxiety inventory (STAI) by Laux

There was no evidence for a statistical relation between anxiety and wish for CRS (*p* = 0.183, chi-squared).

Using the stepwise linear regression analysis taking all measured psychological variables into account, one preserves the following summary (dependent variable: wish for further CRS (*N* = 1652); influencing variables: (constant), problem analysis and coping strategies (FKV) (*F* = 36,739), religiosity and search for sense (FKV) (*F* = 22,917), hedonism (FKV) (*F* = 19,318), depressiveness 2 items (*F* = 15,673) (Table 8)).

**Table 8:** Medical lesions/disorders/deficiencies, mentioned by the patient and further medical data of the questionnaire calculated using Kruskal-Wallis and univariate ANOVA

Lesions/disorders/deficiencies	Univariate analysis (F)	Univariate analysis (significance)	Note
Liquid diet	27,710	< 0.001	
Pap diet	30,739	< 0.001	3^b^
Normal diet	40,705	< 0.001	Control^a^
Gastrogavage	5708	0.017	
PEG	8452	0.004	
Scar formation face or neck	33,779	< 0.001	2^b^
Deficits with eyelid closure	7317	< 0.001	
Suspended labial angle	12,040	< 0.001	
Missing wrinkle formation forehead	3062	0.027	
Salivation leakage out of mouth	19,158	< 0.001	6^b^
Facial muscles ok	48,026	< 0.001	Control^a^
Numb or insentient lower lip	60,432	< 0.001	1^b^
Numb or insentient throat	7145	0.008	
Numb or insentient tongue	8831	0.003	
Hypesthetic region ear	13,286	< 0.001	
Hypesthetic region neck	15,541	< 0.001	
No hypesthetic region	48,730	< 0.001	Control^a^
Pain oral cavity	12,053	0.001	
Pain face	4603	0.032	
Pain temporomandibular joint	20,540	< 0.001	5^b^
Pain other area(s) of the head	8768	0.003	
Pain neck	21,832	< 0.001	4^b^
No pain	12,378	< 0.001	Control^a^
Pain shoulder	17,576	< 0.001	

Using linear stepwise regression calculation, the following are the predictor model results: dependent variable: wish for further CRS; influencing variables: (constant) facial or cervical scarring, fluid/saliva dripping from mouth, hypesthetic lower lip or chin, only fluid diet, gastrostogavage, dropped mouth corner and feeding via nasogastric tube

^a^Control groups: patients got the counter question (no impairment) after questioning for the corresponding impairment

^b^Rank of strength of impairments

Questions on future prospects are psychological variables as well. They are related to the evaluation of appearance and cicatrisation and hence with wishes for CRS. Life quality appraisal and its alteration by cancer are both medical and psychological variables. There was no calculated correlation between life quality and overall tumour size 6 months after surgery (*p* < 0.315, chi-squared). On the contrary, the small group of patients with bigger tumour sizes (pT3) mentioned a higher loss of life quality (*p* < 0.003). The number of patients with pT1 tumour size was unless larger and stated no significant difference in life quality. Referring to the type of treatment, both variables (life quality now and its alteration by cancer) are significantly different (*p* < 0.011 resp. *p* < 0.002).

Psychological assessment furthermore includes the lack of information between physician and patient concerning recurrence. In the questionnaire of physicians, a recurrence was stated 58 times. On the contrary, 272 patients thought they suffered from recurrence and another 106 were not quite sure. There is a significant correlation between the informed consent of surgery, depressiveness (*p* < 0.001; SR 5.2), fear (*p* < 0.001) and life quality at diagnose (*p* < 0.001) of the patient. Those patients who stated to be well informed mainly belonged to the group evaluating a high life quality. Badly informed patients evaluated their future to be more hopeless (*p* < 0.001; SR 5.6). They suffered more often from problems in relationships (*p* < 0.001; SR = 3.8) and frequently avoided public visitations (*p* < 0.001; SR = 3.3).

## Discussion

An important role is played by the question for further CRS in terms of rehabilitation after radical surgery of oral cancer [13]. It is well known that the impairments, deficits and psychological variables are due to this fact. Relations in between those variables are nevertheless inadequately researched and practically implemented [34]. Most oral and maxillofacial surgeons know that most patients do not want to undergo further CRS. However, literature did not describe yet that this population can be as large as 74%. This is maintained among others by the tumour localisation which is contrary to the fact that 90% of our patients had no further CRS. After all, 26% had a strong wish for CRS. This might be explained by patients’ insecurity evaluating medical facts, which can be seen in terms of knowledge on relapse. Physicians diagnosed a relapse in 58 patients, but 272 patients believed to suffer relapse, and another 106 were unsure. A total of 80% were varyingly worried about relapse as Campbell emphasized [35]. Hence, there is an information gap, which could be closed by improving the physician-patient consultation.

No difference was shown between gender and wish for CRS in this survey. Family status on the contrary was important: widowed, divorced and separate as well as single patients mentioned a stronger wish for CRS than those bound to families. Older patients resigned more often than younger ones. Already discussed in literature patients with strong wishes for CRS stated a loss of income more often than those without wish for CRS [7, 36, 37]. The importance of this aspect is emphasized by the fact that loss of income got incorporated in the questionnaire by Rogers [38].

Concerning life quality (LQ), cicatrisation and facial distortion are of major importance, which is conveyed in our study and in literature [3, 8, 16, 35, 39]. Nevertheless, several examiners did not find a reduced life quality in comparison to normal population or other cancer types [40]. Which psychological mechanisms are due to this fact should be urgently resolved, since additional particularizing questions indicate the opposite [41].

Our survey on 19 impairments affirms the suspicion that questionnaires on life quality do not describe the full range of psychological conditions. All 19 impairments are highly correlated to life quality. They change during the period of time after surgery and not only in patients with oral cancer. Hence, appearance itself follows the typical exemplary course of impairments. Pre-operatively, there are no grievances; shortly after surgery, discomfort is at its highest level, and 6 months post-operative values are decreasing. This course of discomfort is often reported in literature for example by Markkanen-Leppänen [42].

Nowadays, oral and maxillofacial surgeons aim for the development and comparison of operational techniques and cosmetically aid to reduce the patients fear of defacement [8, 13, 16, 43–48].

From a list of 19 impairments, each impairment differed significantly in its strength regarding the wish for CRS. As prospected, the most important impairments were appearance and mandibular mobility. Kamstra et al. and Devine et al. mentioned mandibular mobility as main factor [44, 49] as well as Hahn et al. [50]. Furthermore, cervical mobility, articulation of speech and mouth opening are meaningful.

Mobility of the tongue was important to our 410 patients wishing for CRS because of the control of food intake, also described by Toporcov and Antunes [51]. The same holds true for the possibility to open the mouth, described by Weber et al. [52]. Further, 15 impairments do differ between patients with and without wish for CRS, but with less significance (Table 9).

**Table 9:** Differences between patients without (*N* = 1155) and those with (*N* = 410) wish for further CRS

Psychological variables	Chi-square	Patients without wish for CRS (*n* = 1155)	Patients with further wish for CRS: rank/*n* of 410
Questionnaire of illness processing (FKV)	38,788	1094	1/405
Depressive illness coping	19,924	1109	4/408
Hedonisms (FKV)	24,355	1092	3/406
Mistrust und pessimism (FKV)	11,312	1105	408
Internality (KKG)	15,679	1087	6a/401
Overall internality	10,070	1057	6b/391
Emotional support	8818	1111	404
Social burden	19,015	1107	5/404
Depressiveness (2 items)	30,644	1115	2/407
State-Trait-Anxiety (STAI)	5300	1127	406

Medical data implements that the importance of surgery, radiation, dissection and reconstruction is meaningful in developing wishes for further CRS but inadequately researched. And yet they are important preconditions for optimal rehabilitation. There will be no sufficient, satisfying result if there is a great gap between patient’s expectations and medical possibilities [53]. Only an extensive survey among a high number of patients is able to describe the network in between each and every factor. One hundred twenty-five patients were operated once at time of survey. In total, 410 wished for further cosmetic operations. It can be assumed that many of those taking a medical point of view were in no condition for further successful surgery. This fact has to be discussed with the patient, so he/she can feel well treated and life quality remains stable [11, 54, 55].

Among psychosocial variables, the age of patients is important for the wish for CRS. Younger ones more often longed for CRS [56], and additionally, the chance of survival is higher as Goldenberg et al. confirmed in 2009 [57]. After pension, the need for CRS is decreasing, unless it is a premature pension due to the diagnosis of cancer. In this last named case, there was a very strong wish for CRS. Furthermore, patients without partner more often wished for CRS. Some literature describes the coherence of wish for CRS and impairments as, for example, nutrition. Liquid diet increases the wish for further CRS [58, 59]. Equally, pain and hypaesthesia are important for the wish for CRS. Both strongly influence the description of the emotional and functional situation by the patient, yet we were not able to prove a relation to the wish for CRS.

Nevertheless, there is a link to researches on cosmetic surgery in general and on orthognathic surgery. Those surveys show that questions on psychological problems are important for the satisfaction of patients with surgery all the same [5, 60–63]. Hence, we assume that psychological factors play a similar role in patients who underwent surgical treatment of oral cancer. There is a high number of possible measuring tools [14]. The clarification is essential by evaluating the psychological situation of the patient and hence discussing and improving the outcome.

Depressive mood, depressive illness coping and problem analysis are related to further wishes for CRS and hence should be acknowledged by the physician and discussed with the patient. Also, future expectations and evaluation of life quality are of major importance [64–69].

As early as 1980, Sela and Lowental noticed that cancer patients require more than a well-fitting prosthesis for successful rehabilitation [70]. This belief grew throughout the years, but is not yet always realized. Patient management is complicated and depends among others on the patients’ willingness to answer psychological questions [28, 71, 72]. If especially educated nurses or physicians conduct the conversation and psychology is not pronounced, patients may not have those problems. It is furthermore to be examined if patients do understand that their wishes may not be fulfilled all the time and a compromise is to be taken [73, 74]. The ability to get back to work without facial distortion belongs to this set of issues, equalling a signup of full rehabilitation.

## Conclusions

The wish for CRS depends on diverse functional psychosocial and psychological parameters. Hence, it must be issued during conversation to improve rehabilitation. A decision on the medical treatment can be of greater satisfaction if the surgeon knows the patients’ needs and is able to compare them with the medical capabilities. The informed consent between doctor and patient regarding these findings is necessary.

## References

[1] West DW (1977). Social adaptation patterns among cancer patients with facial disfigurements resulting from surgery. Arch Phys Med Rehabil.

[2] Nordlicht S (1979). Facial disfigurement and psychiatric sequelae. N Y State J Med.

[3] Dropkin MJ (1989). Coping with disfigurement and dysfunction after head and neck cancer surgery: a conceptual framework. Semin Oncol Nurs.

[4] Gibson MK, Forastiere AA (2004). Multidisciplinary approaches in the management of advanced head and neck tumors: state of the art. Curr Opin Oncol.

[5] Lyne J, Ephros H, Bolding S (2010). The need for preoperative psychological risk assessment. Oral Maxillofac Surg Clin North Am.

[6] Aarstad AK, Beisland E, Osthus AA, Aarstad HJ (2011). Distress, quality of life, neuroticism and psychological coping are related in head and neck cancer patients during follow-up. Acta Oncol.

[7] Callahan C (2004). Facial disfigurement and sense of self in head and neck cancer. Soc Work Health Care.

[8] Flexen J, Ghazali N, Lowe D, Rogers SN (2012). Identifying appearance-related concerns in routine follow-up clinics following treatment for oral and oropharyngeal cancer. Br J Oral Maxillofac Surg.

[9] Kollbrunner J, Zbaren P, Quack K (2001). Quality of life stress in patients with large tumors of the mouth. 2: dealing with the illness: coping, anxiety and depressive symptoms. HNO.

[10] Kollbrunner J, Zbaren P, Quack K (2001). Quality of life stress in patients with larger tumors of the mouth. A descriptive study of psychosocial effects of illness and primary surgery therapy in 3 parts—1: quantity and quality of life. HNO.

[11] Llewellyn CD, McGurk M, Weinman J (2005). Are psycho-social and behavioural factors related to health related-quality of life in patients with head and neck cancer? A systematic review. Oral Oncol.

[12] Rogers SN, Ahad SA, Murphy AP (2007). A structured review and theme analysis of papers published on ‘quality of life’ in head and neck cancer: 2000–2005. Oral Oncol.

[13] Katre C, Johnson IA, Humphris GM, Lowe D, Rogers SN (2008). Assessment of problems with appearance, following surgery for oral and oro-pharyngeal cancer using the University of Washington appearance domain and the Derriford appearance scale. Oral Oncol.

[14] Kanatas AN, Rogers SN (2010). A systematic review of patient self-completed questionnaires suitable for oral and maxillofacial surgery. Br J Oral Maxillofac Surg.

[15] Cadogan J, Bennun I (2011). Face value: an exploration of the psychological impact of orthognathic surgery. Br J Oral Maxillofac Surg.

[16] Millsopp L, Brandom L, Humphris G, Lowe D, Stat C, Rogers S (2006). Facial appearance after operations for oral and oropharyngeal cancer: a comparison of casenotes and patient-completed questionnaire. Br J Oral Maxillofac Surg.

[17] Nicoletti G, Soutar DS, Jackson MS, Wrench AA, Robertson G (2004). Chewing and swallowing after surgical treatment for oral cancer: functional evaluation in 196 selected cases. Plast Reconstr Surg.

[18] Casabona G, L'Episcopo MR, Di Iorio P, Ciccarelli R, De Bernardis E, Shinozaki H (1994). Interaction between metabotropic receptors and purinergic transmission in rat hippocampal slices. Brain Res.

[19] Hassanein KA, Musgrove BT, Bradbury E (2005). Psychological outcome of patients following treatment of oral cancer and its relation with functional status and coping mechanisms. J Craniomaxillofac Surg.

[20] Lauchlan DT, McCaul JA, McCarron T (2008). Neck dissection and the clinical appearance of post-operative shoulder disability: the post-operative role of physiotherapy. Eur J Cancer Care (Engl).

[21] McGarvey AC, Chiarelli PE, Osmotherly PG, Hoffman GR (2011). Physiotherapy for accessory nerve shoulder dysfunction following neck dissection surgery: a literature review. Head Neck.

[22] Ahlberg A, Nikolaidis P, Engstrom T, Gunnarsson K, Johansson H, Sharp L (2012). Morbidity of supraomohyoidal and modified radical neck dissection combined with radiotherapy for head and neck cancer: a prospective longitudinal study. Head Neck.

[23] Watkins JP, Williams GB, Mascioli AA, Wan JY, Samant S (2011). Shoulder function in patients undergoing selective neck dissection with or without radiation and chemotherapy. Head Neck.

[24] Speksnijder CM, van der Bilt A, Slappendel M, de Wijer A, Merkx MA, Koole R (2013). Neck and shoulder function in patients treated for oral malignancies: a 1-year prospective cohort study. Head Neck.

[25] de Andrade FP, Biazevic MG, Toporcov TN, Togni J, de Carvalho MB, Antunes JL (2012). Discriminant validity of the University of Washington quality of life questionnaire in the Brazilian context. Rev Bras Epidemiol.

[26] Giordano L, Sarandria D, Fabiano B, Del Carro U, Bussi M (2012). Shoulder function after selective and superselective neck dissections: clinical and functional outcomes. Acta Otorhinolaryngol Ital.

[27] Bozec A, Poissonnet G, Chamorey E, Casanova C, Vallicioni J, Demard F (2008). Free-flap head and neck reconstruction and quality of life: a 2-year prospective study. Laryngoscope.

[28] Handschel J, Naujoks C, Hofer M, Kruskemper G (2013). Psychological aspects affect quality of life in patients with oral squamous cell carcinomas. Psychooncology.

[29] Gellrich NC, Suarez-Cunqueiro MM, Bremerich A, Schramm A (2003). Characteristics of oral cancer in a central European population: defining the dentist's role. J Am Dent Assoc.

[30] Gaudry E, Vagg P (1975). Spielberger CD (1975) Validation of the State-Trait Distinction in Anxiety Research. Multivariate Behav Res.

[31] v. Zerssen 1976: Zerssen D (1976) Typus melancholicus" from a psychometric viewpoint (part 1). Z Klin Psychol Psychother 1976;24(3):200–220 1016469

[32] Muthny FA (1996) References for evaluation scales in quality assurance in rehabilitation--6. Assessment of copingprocesses with the Freiburg Questionnaire of Illness Coping. Rehabilitation (Stuttg). 1996;35(2):9–16 8767546

[33] Holder EE (1988). Levi DJ (1988) Mental health and locus of control: SCL-90-R and Levenson's IPC scales. J Clin Psychol.

[34] Adsett CA (1963). Emotional reactions to disfigurement from cancer therapy. Can Med Assoc J.

[35] Campbell BH, Marbella A, Layde PM (2000). Quality of life and recurrence concern in survivors of head and neck cancer. Laryngoscope.

[36] Ng RW, Wei WI (2006). Quality of life of patients with recurrent nasopharyngeal carcinoma treated with nasopharyngectomy using the maxillary swing approach. Arch Otolaryngol Head Neck Surg.

[37] Magne N, Marcy PY, Chamorey E, Guardiola E, Pivot X, Schneider M (2001). Concomitant twice-a-day radiotherapy and chemotherapy in unresectable head and neck cancer patients: a long-term quality of life analysis. Head Neck.

[38] Rogers SN, El-Sheikha J, Lowe D (2009). The development of a patients concerns inventory (PCI) to help reveal patients concerns in the head and neck clinic. Oral Oncol.

[39] Villaret AB, Cappiello J, Piazza C, Pedruzzi B, Nicolai P (2008). Quality of life in patients treated for cancer of the oral cavity requiring reconstruction: a prospective study. Acta Otorhinolaryngol Ital.

[40] Vickery LE, Latchford G, Hewison J, Bellew M, Feber T (2003). The impact of head and neck cancer and facial disfigurement on the quality of life of patients and their partners. Head Neck.

[41] Janni W, Rjosk D, Dimpfl TH, Haertl K, Strobl B, Hepp F (2001). Quality of life influenced by primary surgical treatment for stage I-III breast cancer-long-term follow-up of a matched-pair analysis. Ann Surg Oncol.

[42] Markkanen-Leppanen M, Makitie AA, Haapanen ML, Suominen E, Asko-Seljavaara S (2006). Quality of life after free-flap reconstruction in patients with oral and pharyngeal cancer. Head Neck.

[43] Dziegielewski PT, O'Connell DA, Rieger J, Harris JR, Seikaly H (2010). The lip-splitting mandibulotomy: aesthetic and functional outcomes. Oral Oncol.

[44] Devine, JC, Rogers, SN, McNally, D, Brown, JS, Vaughan, ED (2001) A comparison of aesthetic, functional and patient subjective outcomes following lip-split mandibulotomy and mandibular lingual releasing access procedures*.* Int J Oral Maxillofac Surg 30(3):199–204 10.1054/ijom.2000.0038 11420901

[45] Huang S, Liu HE (2008). Effectiveness of cosmetic rehabilitation on the body image of oral cancer patients in Taiwan. Support Care Cancer.

[46] Yu CH, Chen HM, Hung HY, Cheng SJ, Tsai T, Chiang CP (2008). Photodynamic therapy outcome for oral verrucous hyperplasia depends on the clinical appearance, size, color, epithelial dysplasia, and surface keratin thickness of the lesion. Oral Oncol.

[47] Lorenz KJ, Maier H (2008). Squamous cell carcinoma of the head and neck. Photodynamic therapy with Foscan. HNO.

[48] Wormald R, Donnelly M, Timon C (2009). ‘Minor’ morbidity after parotid surgery via the modified Blair incision. J Plast Reconstr Aesthet Surg.

[49] Kamstra JI, Jager-Wittenaar H, Dijkstra PU, Huisman PM, van Oort RP, van der Laan BF (2011). Oral symptoms and functional outcome related to oral and oropharyngeal cancer. Support Care Cancer.

[50] Hahn TR, Kruskemper G, Enkling N, Kubler NR (2007). On quality of life after surgical therapy of oral cancer—a retrospective multi-center study: the connection between dedentition, denture, quality of life, and dysphagia, and the resulting rehabilitation schemes. Mund Kiefer Gesichtschir.

[51] Toporcov TN, Antunes JL (2006). Restrictions of food intake in patients with oral cancer. Oral Oncol.

[52] Weber C, Dommerich S, Pau HW, Kramp B (2010). Limited mouth opening after primary therapy of head and neck cancer. Oral Maxillofac Surg.

[53] Holloway RL, Hellewell JL, Marbella AM, Layde PM, Myers KB, Campbell BH (2005). Psychosocial effects in long-term head and neck cancer survivors. Head Neck.

[54] List MA, Stracks J, Colangelo L, Butler P, Ganzenko N, Lundy D (2000). How do head and neck cancer patients prioritize treatment outcomes before initiating treatment?. J Clin Oncol.

[55] Kwok HC, Morton RP, Chaplin JM, McIvor NP, Sillars HA (2002). Quality of life after parotid and temporal bone surgery for cancer. Laryngoscope.

[56] Zhang B, Huang HZ, Pan CB, Xu JH, Wang JG, Chen WL Aesthetic and functional radical surgery in young patients with stage one or two tongue cancer: a preliminary report. J Craniomaxillofac Surg 39(3):209–214 10.1016/j.jcms.2010.03.011 20417110

[57] Goldenberg D, Brooksby C, Hollenbeak CS (2009). Age as a determinant of outcomes for patients with oral cancer. Oral Oncol.

[58] Bertrand PC, Piquet MA, Bordier I, Monnier P, Roulet M (2002). Preoperative nutritional support at home in head and neck cancer patients: from nutritional benefits to the prevention of the alcohol withdrawal syndrome. Curr Opin Clin Nutr Metab Care.

[59] Tang JA, Rieger JM, Wolfaardt JF (2008). A review of functional outcomes related to prosthetic treatment after maxillary and mandibular reconstruction in patients with head and neck cancer. Int J Prosthodont.

[60] Hasan JS (2000). Psychological issues in cosmetic surgery: a functional overview. Ann Plast Surg.

[61] Stirling J, Latchford G, Morris DO, Kindelan J, Spencer RJ, Bekker HL (2007). Elective orthognathic treatment decision making: a survey of patient reasons and experiences. J Orthod.

[62] Grossbart TA, Sarwer DB (2003). Psychosocial issues and their relevance to the cosmetic surgery patient. Semin Cutan Med Surg.

[63] Sarwer DB, Crerand CE (2002). Psychological issues in patient outcomes. Facial Plast Surg.

[64] Sherman AC, Simonton S, Adams DC, Vural E, Hanna E (2000). Coping with head and neck cancer during different phases of treatment. Head Neck.

[65] Sherman AC, Simonton S, Adams DC, Vural E, Owens B, Hanna E (2000). Assessing quality of life in patients with head and neck cancer: cross-validation of the European Organization for Research and Treatment of Cancer (EORTC) Quality of Life Head and Neck module (QLQ-H&N35). Arch Otolaryngol Head Neck Surg.

[66] Koga C, Itoh K, Aoki M, Suefuji Y, Yoshida M, Asosina S (2001). Anxiety and pain suppress the natural killer cell activity in oral surgery outpatients. Oral Surg Oral Med Oral Pathol Oral Radiol Endod.

[67] Derks W, Leeuw JR, Hordijk GJ, Winnubst JA (2005). Differences in coping style and locus of control between older and younger patients with head and neck cancer. Clin Otolaryngol.

[68] He G, Liu S (2005). Quality of life and coping styles in Chinese nasopharyngeal cancer patients after hospitalization. Cancer Nurs.

[69] Scharloo M, Baatenburg de Jong RJ, Langeveld TP, van Velzen-Verkaik E, Doorn-op den Akker MM, Kaptein AA (2005). Quality of life and illness perceptions in patients with recently diagnosed head and neck cancer. Head Neck.

[70] Sela M, Lowental U (1980). Therapeutic effects of maxillofacial prostheses. Oral Surg Oral Med Oral Pathol.

[71] Handschel J, Naujoks C, Kubler NR, Kruskemper G (2012). Fear of recurrence significantly influences quality of life in oral cancer patients. Oral Oncol.

[72] Shridharani, SM, Magarakis, M, Manson, PN, Rodriguez, ED (2010) Psychology of plastic and reconstructive surgery: a systematic clinical review*.* Plast Reconstr Surg 126(6):2243–2251 10.1097/PRS.0b013e3181f445ae 21124167

[73] Williams DM, Bentley R, Cobourne MT, Gibilaro A, Good S, Huppa C (2008). The impact of idealised facial images on satisfaction with facial appearance: comparing ‘ideal’ and ‘average’ faces. J Dent.

[74] Sen P, Ross N, Rogers S (2001). Recovering maxillofacial trauma patients: the hidden problems. J Wound Care.

